# Carbon dioxide binding at a Ni/Fe center: synthesis and characterization of Ni(η^1^-CO_2_-κ*C*) and Ni-μ-CO_2_-κ*C*:κ^2^ *O*,*O*′-Fe†
†Electronic supplementary information (ESI) available: Characterization data for **3** and **5**. CCDC 1492006 and 1492007. For ESI and crystallographic data in CIF or other electronic format see DOI: 10.1039/c6sc03450k



**DOI:** 10.1039/c6sc03450k

**Published:** 2016-08-30

**Authors:** Changho Yoo, Yunho Lee

**Affiliations:** a Department of Chemistry , Korea Advanced Institute of Science and Technology (KAIST) , Daejeon 34141 , Republic of Korea . Email: yunholee@kaist.ac.kr ; Fax: +82 42 350 2810 ; Tel: +82 42 350 2814

## Abstract

The degree of CO_2_ activation can be tuned by incorporating a distinct electronic coordination environment at the nickel center. A mononuclear nickel carboxylate species (Ni–CO_2_, **3**) and a dinuclear nickel–iron carboxylate species (Ni–CO_2_–Fe, **5**) were prepared. The structure of **3** reveals a rare η^1^-κ*C* binding mode of CO_2_, while that of **5** shows bridging CO_2_ binding (μ_2_-κ*C*:κ^2^ *O*,*O*′) between the nickel and iron, presented as the first example of a nickel-μ-CO_2_-iron species. The structural analyses of **3** and **5** based on XRD and DFT data reveal a higher degree of CO_2_ activation in **5**, imparted by the additional interaction with an iron ion.

## Abstract 2

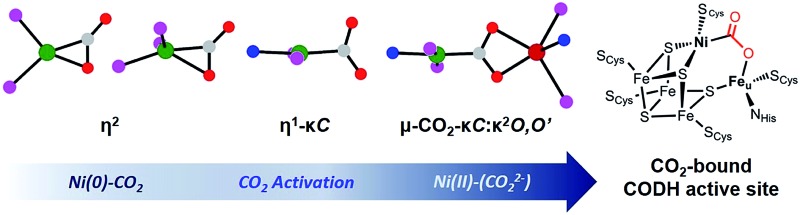
A heterobimetallic Ni-μ-CO_2_-κ*C*:κ^2^ *O*,*O*′-Fe species reminiscent of the CODH active site was synthesized, helping to elucidate the role of the unique iron.

## Introduction

**Scheme 1:**
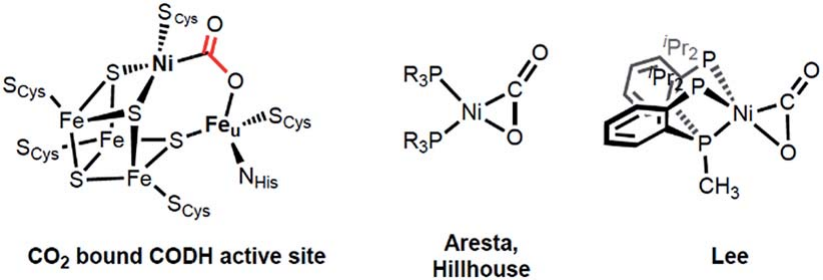
The active site of carbon monoxide dehydrogenase (CODH, left), and 4- and 5-coordinate nickel CO_2_ adducts (right).

Activation of carbon dioxide is currently receiving much attention due to its relevance to environmental and energy related issues.^1^ In the area of transition metal catalyzed reactions, one of the main challenges is selective reduction of CO_2_ to a product such as formate, carbon monoxide, methanol or methane.^2^ In a 2-electron process, the binding mode of the CO_2_ may determine the eventual product formation, *e.g.* formate *vs.* carbon monoxide.^3^ When the initial metal–oxygen interaction occurs to form a metal CO_2_ adduct M-η^1^-CO_2_-κ*O*, subsequent hydride transfer *via* CO_2_ addition to a M–H bond generates a metal-formate species. Alternatively, the metal–carbon bond formation can produce a metallacarboxylate species (M-η^1^-CO_2_-κ*C*), followed by C–O bond cleavage to generate CO. In the latter case, an additional Lewis acid interaction can stabilize the negative charges at the oxygen atoms of the bound CO_2_.^4,5^ Therefore, CO_2_ activation with a bimetallic system can be one way to guide the selectivity of the CO_2_ catalyst and is receiving much attention.^5,6^ In fact, an excellent example of a bimetallic center utilized in an efficient catalytic conversion of CO_2_ can be found in the active site of carbon monoxide dehydrogenase (CODH).^7^ According to recent studies, CO_2_ coordination at a heterobimetallic nickel–iron active site can be found in CODH’s intermediate species, which possesses a Ni-μ-CO_2_-Fe moiety, Scheme 1.^8^ Although X-ray analysis provides a structural snapshot of the CO_2_ reduction sequence, the role of the unique iron ion is currently not well-understood.^7^ Thus, acquiring an understanding of iron assisted CO_2_-nickel coordination is of fundamental interest and is crucial for gaining mechanistic insight into this and other enzymatic reactions.

**Scheme 2:**
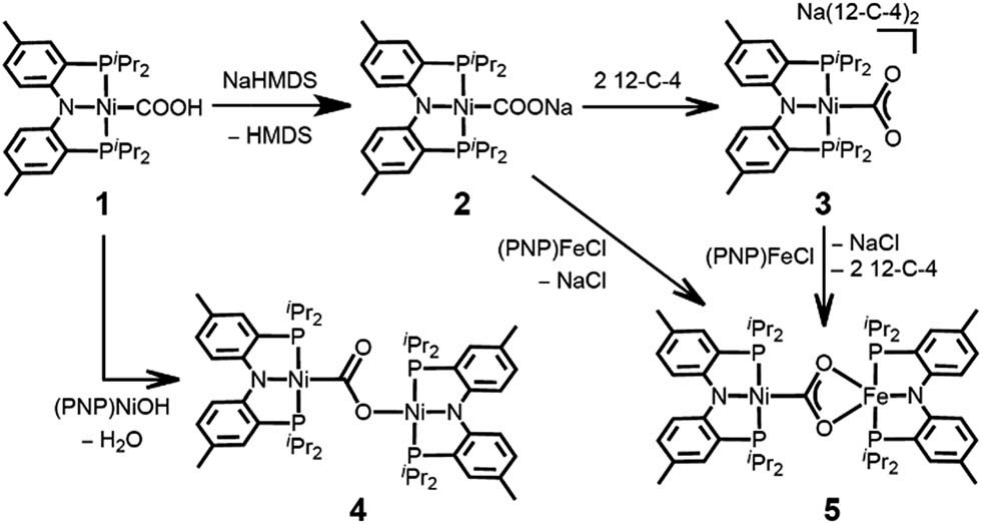
Preparation of mononuclear- and dinuclear-CO_2_ adducts.

In organonickel chemistry, there are few mononuclear Ni-η^2^-CO_2_ adducts possessing both M–C and M–O bonds.^9^ In 1975, Aresta and co-workers reported the first structurally characterized nickel–CO_2_ adduct (PCy_3_)_2_Ni(η^2^-CO_2_), Scheme 1.^9*a*^ An analogous complex, (dtbpe)Ni(η^2^-CO_2_) (dtbpe = 1,2-bis(di-*tert*-butylphosphino)ethane) was recently reported by Hillhouse and co-workers.^9*d*^ More recently, our group reported a similar but unique five-coordinate nickel–CO_2_ adduct (PP^Me^P)Ni(η^2^-CO_2_) (PP^Me^P = PMe(2-P^i^Pr_2_-C_6_H_4_)_2_).^9*f*^ According to its structural analysis, the five coordinate nickel CO_2_ species supported by three neutral P donors has a weak Ni–O bond available for electrophilic attack.^9*f*^ Additionally, by utilizing an anionic tridentate PNP ligand (PNP^–^ = N[2-P^i^Pr_2_-4-Me-C_6_H_3_]_2_^–^), our group reported the nickel hydroxycarbonyl species (PNP)NiCOOH (**1**), (PNP)NiCOONa (**2**) and {(PNP)Ni}_2_-μ-CO_2_-κ^2^ *C*,*O* (**4**), the first examples of Ni–CO_2_ complexes that reveal a Ni–CO_2_-κ*C* binding mode, Scheme 2.^10^ The carboxylate group in these species is stabilized by a Lewis acid such as a proton, sodium or another nickel ion. Our interest then moved to comparing (PP^Me^P)Ni–CO_2_ and (PNP)Ni–CO_2_ to evaluate their fundamental differences in CO_2_ activation. The different geometries favored with a PP^Me^P or PNP ligand affect the identity of the nickel–CO_2_ moiety, which can be Ni(ii)–CO_2_^2–^ or Ni(0)–CO_2_ or an open-shell Ni(i)–CO_2_˙^–^, *vide infra*. Furthermore, by isolating the native nickel–CO_2_ species, we can further study the effect of the second iron ion. Although several nickel carboxylate species are already known, iron has never been introduced synthetically into a Ni–CO_2_ moiety.

Here, we present a nickel carboxylate species {Na(12-C-4)_2_}{(PNP)Ni-η^1^-CO_2_-κ*C*} (**3**), in which the nickel–CO_2_ moiety does not have any Lewis acid interactions. We also prepared a dinuclear nickel–iron carboxylate species (PNP)Ni-μ-CO_2_-κ*C*:κ^2^ *O*,*O*′-Fe(PNP) (**5**), reminiscent of the NiFe-binuclear active site of CODH. This is an unprecedented example of a nickel–iron hetero-bimetallic complex possessing a bridging CO_2_ ligand. The levels of CO_2_ activation in compounds **3** and **5** are compared with other Ni–CO_2_ adducts and the Ni-μ-CO_2_-Fe moiety found in CODH.

## Results and discussion

### Synthesis and characterization of the Ni-η^1^-CO_2_-κ*C* complex

**Fig. 1:**
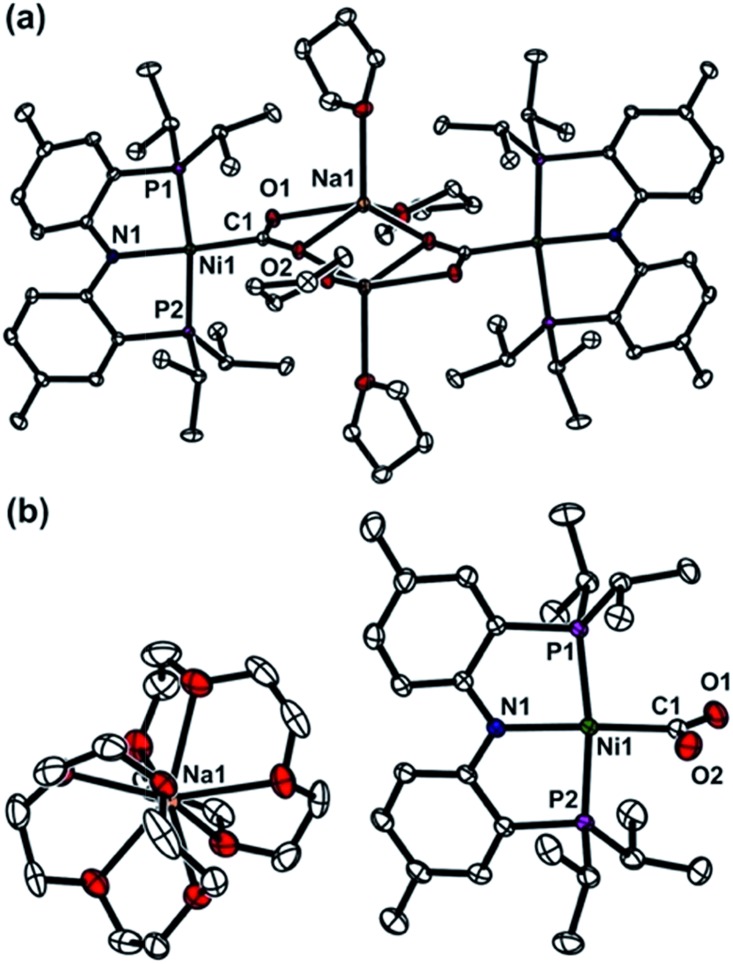
Displacement ellipsoid (50%) representations for (a) (PNP)NiCOONa (**2**) in a dimeric assembly with co-crystallized THF molecules,^11^ and (b) {Na(12-C-4)_2_}{(PNP)Ni-η^1^-CO_2_-κ*C*} (**3**). A co-crystallized 12-crown-4 molecule and hydrogen atoms are omitted for clarity.

**Fig. 2:**
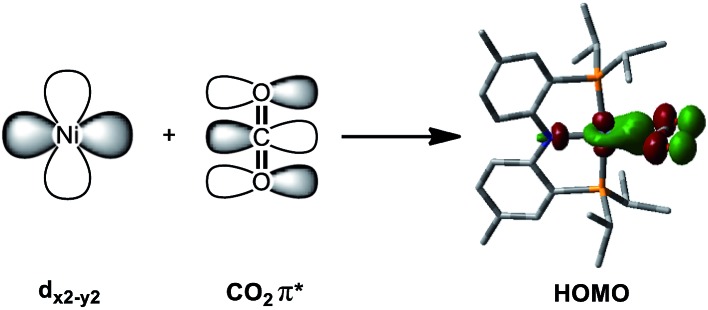
Combination of the Ni d_*x*^2^–*y*^2^_ and CO_2_ π* orbitals provides the DFT calculated HOMO of {Na(12-C-4)_2_}{(PNP)Ni-η^1^-CO_2_-κ*C*} (**3**).

The coordination of a hydroxycarbonyl moiety *via* a Ni–C bond was previously realized at a divalent nickel center supported by a PNP ligand.^10^ Following deprotonation of (PNP)NiCOOH (**1**), its anionic congener (PNP)NiCOONa (**2**) was also prepared and recently reported by our group.^10^ The X-ray structure reveals that two molecules of **2** are oriented to form a pair with ionic interactions with two sodium ions in the crystal lattice, Fig. 1.^11^ The corresponding CO_2_ ligand coordinates to the nickel center in a μ_3_-κ^1^ *C*:κ^2^ *O*,*O*′:κ^1^ *O*′ mode with a Ni–C1 bond distance of 1.882(1) Å. There are additional bonds of the CO_2_ moiety to sodium ions with Na–O bond distances of 2.352(1), 2.217(1) and 2.459(1).^11^ To obtain a sodium-free adduct, 2 equiv. of 12-crown-4 was added to a solution of **2**, resulting in the formation of {Na(12-C-4)_2_}{(PNP)Ni-η^1^-CO_2_-κ*C*} (**3**). The crystal structure of **3** revealed the successful generation of a mononuclear nickel adduct possessing an η^1^-κ*C* coordinated carbon dioxide species with a Ni–C bond distance of 1.911(2) Å, as shown in Fig. 1 and Table 1. The oxidation state of the nickel ion in **3** can be assigned as 2+ based on its similar structural features to previously known nickel(ii) species such as (PNP)NiCOOH (**1**) and {(PNP)Ni}_2_-μ-CO_2_-κ^2^ *C*,*O* (**4**), *vide infra*. The geometry of **3** is square planar (*τ*_4_ = 0.12^12^) with a similar but slightly elongated Ni–C bond distance in comparison to those of **1** and **4** (*d*_Ni–C_ = 1.866(2) and 1.888(2) Å, respectively, Table 1). This is probably due to a lower degree of π back-bonding between the nickel and CO_2_. In fact, π back-donation from the nickel center to a CO_2_ ligand in such nickel carboxylate species is indicated by shorter Ni–C distances (1.858–1.911 Å) than those of the nickel alkyl species (PNP)NiR (R = Me, Et, ^*n*^Pr) (1.963–2.004 Å).^13^ The molecular orbitals generated from DFT calculations also show the presence of π back-donation from the Ni d_*xz*_ to the CO_2_ π* orbital (see ESI†). Due to the absence of Lewis acid interactions in **3**, a lower π-accepting ability of the CO_2_ ligand is expected. Its structural data also revealed that the plane of the CO_2_ ligand is perpendicular to that of the square planar (PNP)Ni moiety. One of the oxygen atoms (*d*_Ni1–O2_ = 2.614(1) Å) is slightly closer to the nickel center than the other (*d*_Ni1–O1_ = 2.776(1) Å), Table 2. These Ni–O distances are much longer than those for other known Ni-η^2^-CO_2_ adducts (1.9–2.2 Å, Table 2), suggesting that neither of the oxygen atoms are bound.^9^ The DFT analysis also supports minimal interaction between the nickel and oxygen atoms (Wiberg index = 0.1358 for Ni1–O2 and 0.1679 for Ni1–O1, see Table 2). The two C–O bond distances are nearly identical (*d*_C1–O1_ = 1.247(2) Å, *d*_C1–O2_ = 1.248(2) Å, Table 1) and slightly shorter than in the analogous carboxylate complexes **2** and **4** (Table 1), due to the absence of a Lewis acid, Na or Ni. According to the DFT analysis, the HOMO of **3** possesses contributions from both a nickel d_*x*^2^–*y*^2^_ orbital and a CO_2_ π* orbital, see Fig. 2. Due to additional electron density from CO_2_^2–^ being shifted to the nickel, the CO_2_ moiety is slightly oxidized compared to the sp^2^ hybridized carboxylate ligands found in **2** and **4**. The larger O–C–O angle (128.4(2)°) of **3** compared to others (124.0(1) and 123.7(2)°) also supports this electronic feature, *vide infra*. Although an η^1^-κ*C* CO_2_ coordination mode has been proposed for many CO_2_ reduction strategies,^2,3^ the only example of a crystallographically identified metal η^1^-κ*C* CO_2_ complex is a rhodium CO_2_ adduct, Rh(CO_2_)(Cl)(diars)_2_ (diars = *o*-phenylene-bis(dimethylarsine)), reported by the Herskovitz group.^14^ According to their C–O bond distances (1.20(2) and 1.25(2) Å) and O–C–O angle (126(2)°), the CO_2_ moiety in **3** shares similar structural and electronic features. Thus, compound **3** is a unique example possessing η^1^-κ*C* CO_2_ binding, since such a binding mode is unknown for 1^st^ row transition metals and is rare in structurally characterized metal–CO_2_ adducts.

**Table 1:** Selected bond distances and angles for the nickel carboxylate species **1**, **2**, **3**, **4** and **5**, and CO_2_-bound CODH

	**1** ^10^	**2** ^11^	**3**	**4** ^10^	**5**	CODH^8*b*^
*d*_Ni–C_ (Å)	1.866(2)	1.882(1)	1.911(2)	1.888(2)	1.858(1)	1.805(31)
*d*_M–O_ (Å)	—	2.352(1)^*a*^	—	1.897(2)^*b*^	2.204(1)^*c*^	2.030(18)^*c*^
		2.217(1)^*a*^			2.066(1)^*c*^	
		2.459(1)^*a*^				
*d*_C–O_ (Å)	1.269(3)	1.260(1)	1.247(2)	1.240(3)	1.269(2)	1.298(30)
	1.313(3)	1.271(1)	1.248(2)	1.296(3)	1.289(2)	1.316(30)
Δ*d*_C–O_ (Å)	0.044	0.011	0.001	0.056	0.020	0.018
∠O–C–O (°)	119.6(2)	124.0(1)	128.4(2)	123.7(2)	116.5(1)	117.2(26)

^*a*^M = Na.

^*b*^M = Ni.

^*c*^M = Fe.

**Table 2:** Selected physical parameters and bond indices from the natural bond orbital analysis

	Ni(PCy_3_)_2_(η^2^-CO_2_)^9*a*^	(dtbpe)Ni(η^2^-CO_2_)^9*d*^	(PP^Me^P)Ni(η^2^-CO_2_)^9*f*^	**3**	**5**
**Structural parameters**					
*d*_Ni–C_ (Å)	1.84(2)	1.868(2)	1.904(1)	1.911(2)	1.858(1)
*d*_Ni–O_ (Å)	1.99(2)	1.904(2)	2.191(1)	2.614(1)	2.718(1)
				2.776(1)	2.792(1)
*d*_C–O_ (Å)	1.17(2)	1.200(3)	1.218(2)	1.248(2)	1.269(2)
	1.22(2)	1.266(3)	1.252(2)	1.247(2)	1.289(2)
∠O–C–O (°)	133	138.0(2)	135.1(1)	128.4(2)	116.5(1)
*ν*_CO_2__ (cm^–1^)	1740	1724	1682	1620	1510

**Wiberg bond indices** ^*a*^					
Ni–C	—	0.5766	0.5286	0.6143	0.6277
Ni–O	—	0.4300	0.3117	0.1679	0.0798
				0.1358	0.0644
C–O	—	1.6927	1.6384	1.5112	1.3993
		1.4080	1.4701	1.4949	1.2933

^*a*^Wiberg bond indices were calculated using single-point calculations, for which geometries were obtained from the XRD data.

### Synthesis and characterization of the heterobimetallic nickel–iron CO_2_ complex

**Fig. 3:**
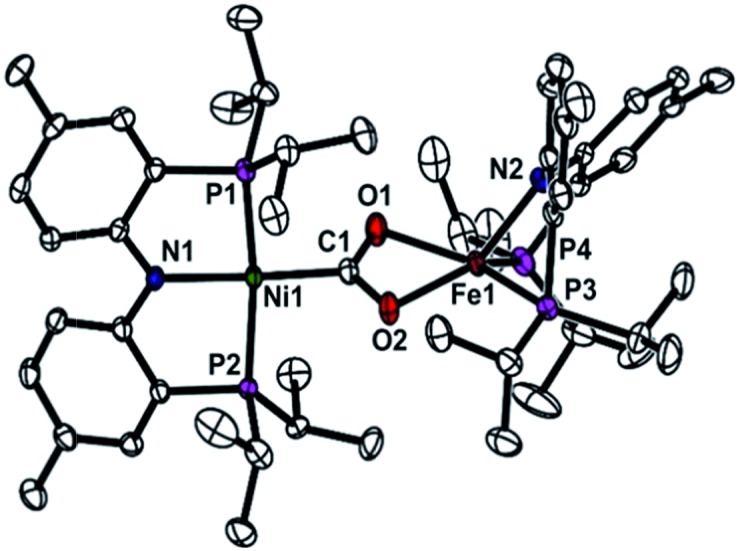
Displacement ellipsoid (50%) representation for (PNP)Ni-μ-CO_2_-κ*C*:κ^2^ *O*,*O*′-Fe(PNP) (**5**). Hydrogen atoms are omitted for clarity.

To gain a better understanding of the role of the second metal ion in the CODH active site, we prepared a heterobimetallic nickel–iron carboxylate species possessing a Ni–CO_2_–Fe fragment by addition of {(PNP)Fe}^+^ to a Ni-η^1^-CO_2_-κ*C* species. To a yellow solution of {Na(12-C-4)_2_}{(PNP)Ni-η^1^-CO_2_-κ*C*} (**3**) in toluene, a purple solution of (PNP)FeCl was added. The immediate formation of a new orange species (PNP)Ni-μ-CO_2_-κ*C*:κ^2^ *O*,*O*′-Fe(PNP) (**5**) was confirmed, using the ^1^H NMR spectrum, from the absence of peaks for **3** and (PNP)FeCl and the presence of new paramagnetically shifted signals. The same product was also prepared by substitution of the sodium ion of (PNP)NiCOONa (**2**) with (PNP)FeCl. The solid-state structure of **5** clearly revealed a dinuclear nickel–iron complex with a bridging CO_2_ ligand in the μ_2_-κ*C*:κ^2^ *O*,*O*′ mode (Fig. 3). The Ni and Fe ions are separated by a distance of 4.3690(3) Å. The two C–O bond distances are 1.269(2) and 1.289(2) Å, revealing that a significant elongation has occurred due to the iron interaction compared to **3** (Table 1). The bond distances between the iron and both oxygen atoms are 2.204(1) and 2.066(1) Å. The nickel center possesses a square planar geometry (*τ*_4_ = 0.10^12^). The geometry around the iron is distorted square pyramidal (*τ* = 0.13,^15^ Fig. 3). The O1–C1–O2 angle (116.5°) reflects the sp^2^ hybridization of the carboxylate ligand in **5**. In fact, recent crystallographic data of CODH at atomic resolution (*d*_min_ = 1.03 Å) revealed that the bound CO_2_ molecule (∠O–C–O = 117.2(26)°) is a carboxylate anion (CO_2_^2–^).^8b,8c^ Regarding the similarity between these angles, the carboxylate moiety in **5** might be close to CO_2_^2–^. The asymmetric vibration for CO_2_ observed at 1510 cm^–1^, which is similar to that observed for the dinickel carboxylate species (**4**) at 1518 cm^–1^, also indicates a reduced state of CO_2_. The effective magnetic moment of **5** was determined using the Evans' method (*μ*_eff_ = 4.95 *μ*_B_ in C_6_D_6_), which indicated an *S* = 2 spin state.^16^ According to DFT calculations, most of the spin density is located on the iron center (see ESI†). For CODH, the unique iron, Fe_u_, was assigned as a high spin iron(ii) (ferrous component II, FCII) using Mössbauer spectroscopy,^17^ and a low-spin nickel(ii) was demonstrated using X-ray absorption spectroscopy (XAS).^18^ The current structural and spectroscopic analyses suggest that **5** might share a similar electronic structure to that found in CODH. Gibson classified the μ_2_-κ*C*:κ^2^ *O*,*O*′ binding modes of CO_2_ into two types according to the difference between the two C–O distances.^19^ Due to the two similar C–O distances of the CO_2_ moiety, compound **5** (Δ*d*_C–O_ = 0.020 Å) can be assigned as a class I complex.^20^ In the dinickel CO_2_ species (**4**), the CO_2_ molecule is coordinated in a μ_2_-κ*C*:κ*O* mode with the absence of a Ni2–O2 interaction (*d*_Ni2–O2_ = 3.14(7)) and two different C–O bond distances (Δ*d*_C–O_ = 0.056 Å). In CODH, CO_2_ is coordinated in a μ_2_-κ*C*:κ*O* fashion between the nickel and iron ions, but the two C–O bond distances are quite comparable (Δ*d*_C–O_ = 0.018 Å), akin to the μ_2_-κ*C*:κ^2^ *O*,*O*′ mode. This might be due to hydrogen bonding with the protein matrix, since both the CO_2_ oxygens are hydrogen bonded to His93 and Lys563, respectively.^8^

Compound **5** is the first example of a dinuclear nickel–iron–CO_2_ complex. While dinuclear CO_2_ complexes mostly employ 2^nd^ and 3^rd^ row transition metals,^19^ several bimetallic iron carboxylates (Fe–CO_2_–M, M = Ti, Zr, Sn, Re) have been reported.^5a,5c–e,21^ However, such complexes typically possess an Fe–C bond rather than an Fe–O bond with CO_2_. There have been numerous examples of nickel–iron bimetallic complexes reported for synthetic model studies of NiFe hydrogenase,^22^ but a bimetallic complex possessing a Ni-μ-CO_2_-Fe moiety closely related to CODH chemistry is not known. The Holm group constructed a series of [NiFe_3_S_4_] cubanes as structural model complexes for the [NiFe_4_S_4_] core in CODH.^23^ However, the installation of an iron species corresponding to the Fe_u_ in CODH and reactions involving CO and CO_2_ have not yet been investigated. More recently, the Holm group also reported a bimetallic complex containing nickel and iron supported by a binucleating macrocycle.^24^ With respect to CODH chemistry, bridging hydoroxo, cyanido and formato species have been generated, however, a Ni-μ-CO_2_-Fe fragment had not yet been isolated.

### Activation of CO_2_ in **3** and **5**

Previously known 4-coordinate Ni–CO_2_ complexes possessing an η^2^-CO_2_ binding mode have a formally zero-valent nickel center, Scheme 1.^9^ This suggests limited CO_2_ activation in such species. Interestingly, the 5-coordinate nickel CO_2_ adduct (PP^Me^P)Ni(CO_2_) also has a similar level of activation of CO_2_ based on the C–O bond distances and the O–C–O angle, Table 2. Regarding CO_2_ binding and activation, {Na(12-C-4)_2_}{(PNP)Ni-η^1^-CO_2_-κ*C*} (**3**) is a unique example. It is striking that a neutral pincer-type ligand PP^Me^P (PP^Me^P = PMe[2-P^i^Pr_2_-C_6_H_4_]_2_) favors 5-coordinate η^2^-CO_2_ coordination at a single nickel center, while **3** remains as a 4-coordinate species with η^1^-CO_2_ coordination.^9*f*^ Although the total number of Ni d-electrons and CO_2_ π*-electrons in both **3** and (PP^Me^P)Ni(CO_2_) is the same, (PP^Me^P)Ni(CO_2_) can be considered as formally Ni(0)–(CO_2_) while **3** can be better described as a Ni(ii)–(CO_2_^2–^) species. The asymmetric CO_2_ stretching frequency for **3** is significantly shifted to a lower vibration, 1620 cm^–1^, compared to those of the Ni-η^2^-CO_2_ complexes (Table 2), which is evidence of a reduced CO_2_ moiety in **3**.^9^ This may be due to the influence of the *trans* atom: an anionic amide nitrogen *vs.* a neutral phosphorus atom. The anionic nitrogen in **3** electrostatically favors a divalent nickel center, while the neutral π-acidic P atom in the PP^Me^P ligand favors a Ni(0) center. In fact, the PNP ligand typically stabilizes a square planar geometry while the PP^Me^P ligand favors a pseudo-tetrahedral geometry. Thus, **3** prefers to accommodate a divalent nickel center while (PP^Me^P)Ni(CO_2_) prefers Ni(0). However, the reduction state of the CO_2_ moiety in **3** is a little ambiguous according to the O–C–O angle. The O–C–O angle in **3** of 128.4(2)° is larger than those of an ideal sp^2^ hybridized carbon (120°) and the other nickel(ii) carboxylate species **1**, **2** and **4** (119.6(2)°, 124.0(1)° and 123.7(2)°, respectively, Table 1). The O–C–O angle of a CO_2_ radical anion (CO_2_˙^–^) is suggested to be 133°,^25^ which is fairly similar to those of the previously reported Ni-η^2^-CO_2_ complexes, Table 2. Thus, the geometry of the CO_2_ moiety in **3** may be thought of as being between a CO_2_ radical anion and a carboxylate.

Upon addition of iron to compound **3**, the CO_2_ is further reduced to carboxylate (CO_2_^2–^). The C–O bond distances and O–C–O angle in **5** clearly show a 2-electron reduced state of the CO_2_ moiety, Table 2. This was also indicated by the asymmetric CO_2_ vibration observed at 1510 cm^–1^, which is significantly lower than those of other CO_2_ species and **3**. The Wiberg bond indices nicely agree with the bond distances, Table 2. These analyses of a series of nickel–CO_2_ compounds demonstrate how the degree of CO_2_ activation can be tuned by incorporating a distinct electronic coordination environment at the metal center, and may have parallels to the efficient CO_2_ conversion found in CODH.

In order to study further activation of the bound CO_2_ *via* C–O bond cleavage, protonation of **3** and **5** was attempted. Our group previously reported that reversible C–O bond cleavage/formation occurs with a nickel hydroxycarbonyl species (**1**).^10^ From reaction of **3** with 1 equiv. of HBF_4_·Et_2_O, a nickel hydroxycarbonyl species (**1**) was produced and isolated with a 74% yield. A similar reaction of **3** with 2 equiv. of HBF_4_·Et_2_O resulted in the formation of a carbonyl species {(PNP)NiCO}{BF_4_} in 75% yield, revealing that two sequential protonations can occur with compound **3** possessing a Ni-η^1^-CO_2_-κ*C* moiety, which are key steps in the transformation of CO_2_ to CO. Although protonation of compound **5** seems to produce **1** and {(PNP)NiCO}{BF_4_}, unfortunately, their yields were not clear due to thermal decomposition of **5** and the generation of multiple products. Demetallation of the iron seems to be one of the decomposition processes.

## Conclusions

In conclusion, the generation of unprecedented nickel–carbon dioxide adducts possessing a Ni–C bond accommodated by a (PNP)Ni scaffold was accomplished. A mononuclear CO_2_ adduct {Na(12-C-4)_2_}{(PNP)Ni-η^1^-CO_2_-κ*C*} (**3**) and a dinuclear nickel–iron carboxylate species (PNP)Ni-μ-CO_2_-κ*C*:κ^2^ *O*,*O*′-Fe(PNP) (**5**) were synthesized successfully. While the solid state structure of **3** revealed a rare η^1^-κ*C* binding mode, compound **5** was structurally characterized to reveal a unique class I type μ_2_-κ*C*:κ^2^ *O*,*O*′ binding mode. This heterobimetallic CO_2_ adduct is the first example of a nickel–iron carboxylate species, of which the structural and electronic features are reminiscent of those of the Ni-μ-CO_2_-Fe fragment found in the C-cluster of CODH. Comparison of the η^1^-CO_2_-κ*C* species **3** and dinuclear Ni-μ-CO_2_-Fe species **5** with previously reported Ni–CO_2_ adducts suggested that the CO_2_ ligand can be stabilized and activated by interaction with the second metal. Protonation of **3** produces a nickel carbonyl species {(PNP)NiCO}{BF_4_} *via* C–O bond cleavage, while the reactivity of **5** is limited. Further studies on incorporating a stable iron species and the subsequent reactivity toward protonation are currently underway.
