# Supplementary material for: Carbon dioxide binding at a Ni/Fe center: synthesis and characterization of Ni(η1-CO2-κC) and Ni-μ-CO2-κC:κ2 O,O′-Fe
Source: Chem Sci. 2016 Aug 30;8(1):600–5. doi: 10.1039/c6sc03450k (PMC5458731; doi:10.1039/c6sc03450k)
Supplement: Supplementary file 1 [file SC-008-C6SC03450K-s001.pdf]

Electronic Supplementary Material (ESI) for Chemical Science.
This journal is © The Royal Society of Chemistry 2016

Electronic Supplementary Information for

Carbon Dioxide Binding at a Ni/Fe Center; Synthesis and Characterization of (PNP)Ni(η^1-CO_2-κ*C*) and (PNP)Ni-μ-CO_2-κ*C*:κ^2*O,O'*-Fe(PNP)

Changho Yoo and Yunho Lee*
Department of Chemistry, Korea Advanced Institute of Science and Technology
Daejeon 34141, Republic of Korea

Contents

Experimental Section
Figure S1. ^1H NMR spectrum of {Na(12-C-4)$_2$}{(PNP)Ni-η^1-COO-κ*C*} (**3**).
Figure S2. ^{13}C NMR spectrum of {Na(12-C-4)$_2$}{(PNP)Ni-η^1-COO-κ*C*} (**3**).
Figure S3. ^{31}P NMR spectrum of {Na(12-C-4)$_2$}{(PNP)Ni-η^1-COO-κ*C*} (**3**).
Figure S4. ^1H NMR spectrum of (PNP)Ni-μ-CO_2-κ*C*:κ^2*O,O'*-Fe(PNP) (**5**).
Figure S5. Solid-state structure of **3**.
Table S1. Selected bond distances and angles for **3**.
Figure S6. Solid-state structure of **5**.
Table S2. Selected bond distances and angles for **5**.
Figure S7. UV-Vis spectra of **3**.
Figure S8. UV-Vis spectra of **5**.
Figure S9. IR spectra of **1**, **2** and **3**.
Figure S10. IR spectra of **4** and **5**.
Figure S11. DFT calculated HOMO for **3**.
Figure S12. Electronic structures for **3** and **1.**
Table S3. Selected Wiberg bond indices for (dtbpe)Ni(CO_2), ($PP^{Me}P$)Ni(CO_2), **3**, **1** and **5**.
Figure S13. Mülliken atomic spin density plot derived from the DFT calculation of **5**.
References

Experimental Section

General Considerations. All manipulations were carried out using standard Schlenk or glovebox techniques under a N_2 atmosphere. Solvents were deoxygenated and dried by thoroughly sparging with Ar gas followed by passage through an activated alumina column. Solvents were tested with a standard purple solution of sodium benzophenone ketyl in tetrahydrofuran in order to confirm effective oxygen and moisture removal. All reagents were purchased from commercial vendors and used without further purification unless otherwise stated. $\{(PNP)NiCOONa\}_2$•THF (**2**)[1] and (PNP)FeCl[2] were prepared according to literature procedures. Elemental analyses were carried out on a Thermo Scientific FLASH 2000 series instrument at the KAIST Analysis Center for Research Advancement. Deuterated solvents were purchased from Euriso-top, degassed, and dried over activated 4-Å molecular sieves prior to use.

X-ray Crystallography. The diffraction data of $\{Na(12\text{-}C\text{-}4)_2\}$\{(PNP)NiCOO\} and (PNP)Ni-CO_2-Fe(PNP) was collected on a Bruker D8 QUEST. A suitable size and quality of crystal was coated with Paratone-*N* oil and mounted on a Dual-Thickness MicroLoops LD purchased from MiTeGen. The data were collected with graphite-monochromated MoKα radiation (λ = 0.71073 Å) under a stream of N_2 (g) at 120 K. Cell parameters were determined and refined by SMART program.[3] Data reduction was performed using SAINT software.[4] An empirical absorption correction was applied using the SADABS program.[5] The structures were solved by direct methods and all nonhydrogen atoms were subjected to anisotropic refinement by full-matrix least-squares on F^2 by using the SHELXTL/PC package.[6] Unless otherwise noted, hydrogen atoms were placed at their geometrically calculated positions and refined riding on the corresponding carbon atoms with isotropic thermal parameters.

Spectroscopic Measurements. A Bruker AVHD-400 spectrometer was used to measure ^1H, ^{13}C and ^{31}P NMR. The chemical shifts for ^1H and ^{13}C NMR spectra were quoted in part per million referenced to residual solvent peaks. The following abbreviations were used to describe peak splitting patterns when appropriate: s = singlet, d = doublet, t = triplet, q = quartet, m = multiplet, br s = broad singlet. Coupling constants, *J*, were reported in hertz unit (Hz). ^{31}P NMR spectra were decoupled by broad band proton decoupling. The chemical shifts for ^{31}P NMR spectra were quoted in part per million (ppm) referenced to external phosphoric acid as 0.0 ppm. Solution magnetic moments were determined by the Evans' method.[7] UV-vis spectra were measured by an Agilent Cary 60 UV-vis spectrophotometer using a 1-cm two-window quartz cell sealed with a screw-cap purchased from Hellma Analytics (117.100-QS). Infrared spectra were recorded in KBr pellet by a Bruker VECTOR 33. Frequencies are given in reciprocal centimeters (cm^{-1}) and only selected absorbances were reported.

Computational details. Single-point calculation and NBO analysis for **1**, **3**, **5**, (dtbpe)Ni(CO_2)[8a] and ($PP^{Me}P$)Ni(CO_2)[8b] were run on the Gaussian09[9] with B3LYP density functional theory. The lanl2dz basis set associated with effective core potential was used for describing a nickel atom and 6-31+G** basis set was used for all other atoms. Geometries were obtained from XRD data.

Synthesis of {Na(12-C-4)$_2$}{(PNP)Ni-η^1-COO-κ*C*} (3). To an orange solution of {(PNP)NiCOONa}$_2$•THF (200 mg, 0.169 mmol) in 15 mL THF, 12-crown-4 (120 mg, 0.681 mmol) was added. The reaction mixture was stirred for 10 min at room temperature. The solution was filtered through Celite and the volatiles were removed by vacuum. The resulting product {Na(12-C-4)$_2$}{(PNP)Ni-η^1-COO-κ*C*} (**3**, 267 mg, 0.294 mmol, 87.0%) was isolated as an yellow solid after washing with pentane and drying under vacuum. ^{1}H NMR (400 MHz, C_6D_6) δ 7.83 (d, J = 8.3 Hz, 2H), 7.14 (s, 2H), 6.91 (d, J = 8.0 Hz, 2H), 3.48 (s, 32 H), 2.56–2.46 (m, 4H), 2.29 (s, 6H), 1.48 (q, J = 7.7 Hz, 12H), 1.32 (q, J = 6.9 Hz, 12H). ^{13}C NMR (400 MHz, C_6D_6) δ 197.25, 161.38, 132.38, 131.59, 122.82 (t, J = 16.0 Hz), 122.23, 114.67, 68.87, 23.31, 20.82, 19.21, 17.99. ^{31}P NMR (162 MHz, C_6D_6) δ 36.6. Anal. Calcd. for $C_{53}H_{80}FeN_2NiO_2P_4$: C, 56.96; H, 8.00; N, 1.54. Found: C, 56.93; H, 7.86; N, 1.72. UV-Vis [THF, nm (L mol^{-1} cm^{-1})]: 650 (290), ~430 (sh, 1500), ~390 (sh, 2500), 347 (20000), 315 (22000). IR (KBr pellet, cm^{-1}): ν_{Ar} = 1595, $\nu_{C=O}$ = 1622. Crystals suitable for X-ray diffraction were obtained by slow evaporation of an ether solution.

Reaction of 3 with 1 equiv. of HBF$_4$•Et$_2$O. To a yellow solution of {Na(12-C-4)$_2$}{(PNP)NiCOO} (**3**, 30 mg, 0.033 mmol) in 5 mL THF, HBF$_4$•Et$_2$O (4.5 μL, 5.3 mg, 0.033 mmol) was added using a microsyringe at –78 °C. The reaction mixture was stirred for 30 min at room temperature. The resulting solution was filtered through Celite and all volatiles were removed under vacuum. The resulting product (PNP)NiCOOH (**1**, 13 mg, 0.024 mmol, 74%) was isolated as a yellow solid after washing with toluene and drying under vacuum. The identity of the product was confirmed by its ^{1}H and ^{31}P NMR spectrum.[1]

Reaction of 3 with 2 equiv. of HBF$_4$•Et$_2$O. To a yellow solution of {Na(12-C-4)$_2$}{(PNP)NiCOO} (**3**, 30 mg, 0.033 mmol) in 5 mL THF, HBF$_4$•Et$_2$O (9.0 μL, 11 mg, 0.066 mmol) was added using a microsyringe at –78 °C. The reaction mixture was stirred for 30 min at room temperature resulting in a color change to green. All volatiles were removed under vacuum. After dissolved in toluene a green solution was filtered through Celite and volatiles were removed under vacuum. From layering pentane over a concentrated THF solution, the resulting product {(PNP)NiCO}{BF$_4$} (15 mg, 0.025 mmol, 75%) was obtained as a green powder after washing with additional pentane and dried under vacuum. The identity of the product was confirmed by its ^{1}H and ^{31}P NMR spectrum.[1]

Synthesis of (PNP)Ni-μ-CO$_2$-κ*C*:κ^2*O,O'*-Fe(PNP) (5) from 2. To a yellow solution of {(PNP)NiCOONa}$_2$•THF (**2**, 120 mg, 0.102 mmol) in 20 mL of toluene, a purple solution of (PNP)FeCl (106 mg, 0.204 mmol) in 10 mL toluene was added dropwise at –78 °C resulting in an immediate color change to orange. The reaction mixture was stirred at room temperature for 30 min and filtered through Celite. The volatiles were removed under vacuum. The resulting red sticky solid was dissolved in benzene and lyophilized. The product (PNP)Ni-μ-CO$_2$-κ*C*:κ^2*O,O'*-Fe(PNP) (**5**, 106 mg, 0.104 mmol, 51.2%) was isolated as an yellow powder after washing with pentane and drying under vacuum. ^{1}H NMR (400 MHz, C_6D_6) δ 48.00 (br s, $\nu_{1/2}$ = 75 Hz), 38.90 (br s, $\nu_{1/2}$ = 201 Hz), 37.18 (br s, $\nu_{1/2}$ = 134 Hz), 12.90 (br s, $\nu_{1/2}$ = 44 Hz), 10.28 (br s, $\nu_{1/2}$ = 55 Hz), 9.43 (br s, $\nu_{1/2}$ = 40 Hz), 4.51 (br s, $\nu_{1/2}$ = 163 Hz), 3.91 (br s, $\nu_{1/2}$ = 38 Hz), 3.26 (br s), 2.11 (br s, $\nu_{1/2}$ = 62 Hz), 1.23 (br s, $\nu_{1/2}$ = 84 Hz), 0.87 (br s, $\nu_{1/2}$ = 40 Hz), –0.09 (br s, $\nu_{1/2}$ = 418 Hz), –2.19 (br), –6.34 (br s, $\nu_{1/2}$ = 523 Hz), –14.19 (br s, $\nu_{1/2}$ = 495 Hz). μ_{eff}: 4.95 μB (C_6D_6, 25 °C, Evans' method). Anal. Calcd. for $C_{53}H_{80}FeN_2NiO_2P_4$•1/2(benzene): C, 63.77; H, 7.93; N, 2.66. Found: C, 63.71; H, 7.74; N, 2.52. UV-Vis [THF, nm (L mol^{-1} cm^{-1})]: ~430 (sh, 330), 346

(29000), 309 (31000). IR (KBr pellet, cm^{-1}): ν_{Ar} 1595, ν_{CO2} 1510. Crystals suitable for X-ray diffraction were obtained by slow evaporation of an ether solution.

Synthesis of (PNP)Ni-μ-CO_2-κ*C*:κ^2*O,O'*-Fe(PNP) (5) from 3. To a yellow solution of {Na(12-C-4)$_2$}{(PNP)NiCOO} (**3**, 208 mg, 0.229 mmol) in 20 mL of toluene, a purple solution of (PNP)FeCl (119 mg, 0.229 mmol) in 10 mL toluene was added dropwise at –78 °C resulting in an immediate color change to orange. The reaction mixture was stirred at room temperature for 30 min and filtered through Celite. The volatiles were removed under vacuum. The resulting red sticky solid was dissolved in benzene and lyophilized. The resulting product (PNP)Ni-CO_2-Fe(PNP) (106 mg, 0.104 mmol, 45.6%) was isolated as a yellow powder after washing with pentane and drying under vacuum. The identity of the product was confirmed by its ^1H NMR spectrum.

Figure S1. ^{1}H NMR spectrum of {Na(12-C-4)$_2$}{(PNP)Ni-η^1-COO-κ*C*} (**3**) in C_6D_6 at room temperature.

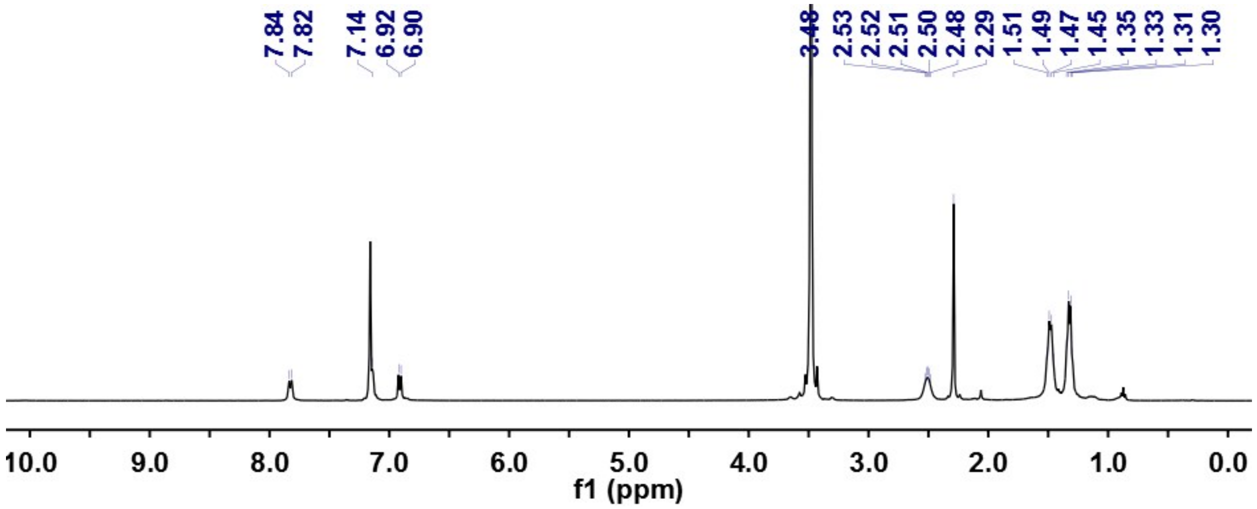

Figure S2. ^{13}C NMR spectrum of {Na(12-C-4)$_2$}{(PNP)Ni-η^1-COO-κ*C*} (**3**) in C_6D_6 at room temperature.

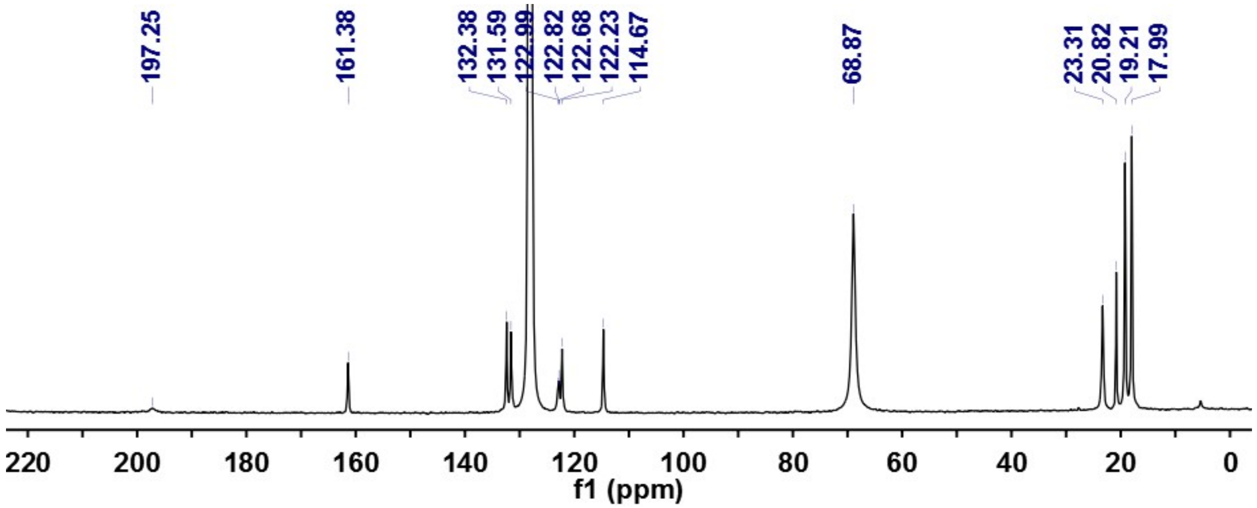

Figure S3. ^{31}P NMR spectrum of {Na(12-C-4)$_2$}{(PNP)Ni-η^1-COO-κ*C*} (**3**) in C_6D_6 at room temperature.

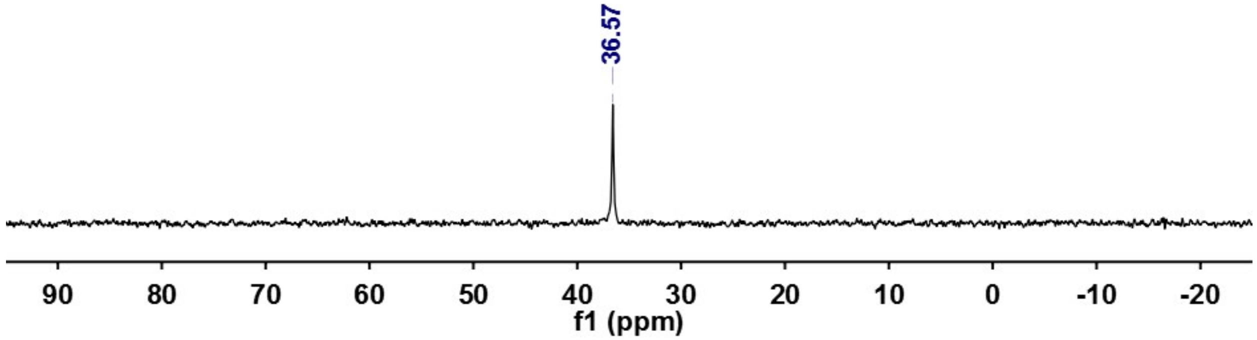

Figure S4. ^{1}H NMR spectrum of (PNP)Ni-μ-CO_2-κ*C*:κ^2*O,O'*-Fe(PNP) (**5**) in C_6D_6 at room temperature.

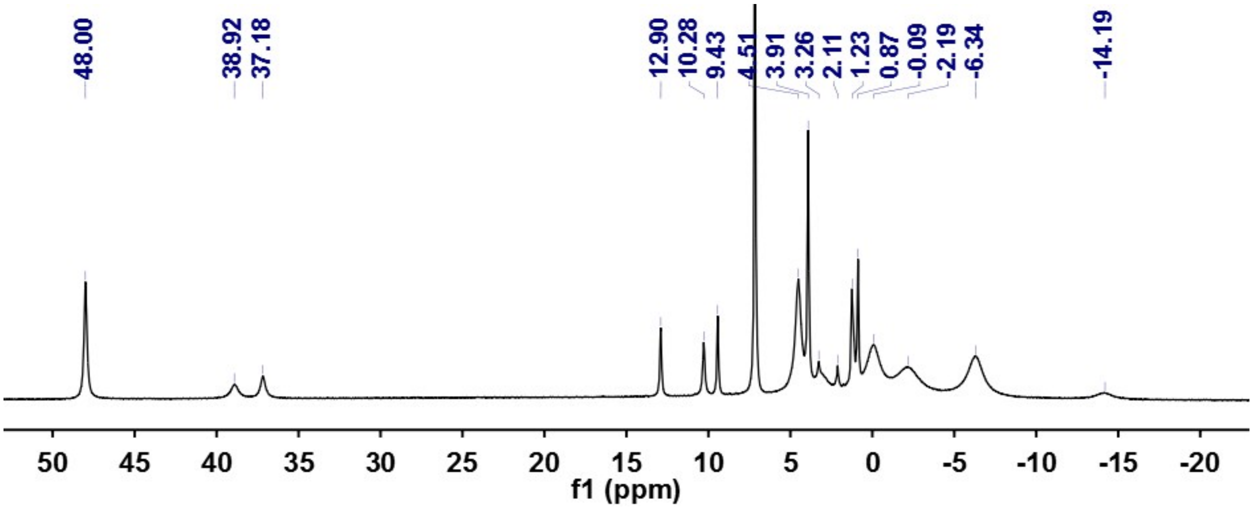

Figure S5. Solid-state structure of {Na(12-C-4)$_2$}{(PNP)Ni-η^1-COO-κ*C*} (**3**). One co-crystallized free 12-crown-4 molecule and hydrogen atoms are omitted for clarity. The 12-crown-4 molecules are disordered over two distinct positions. For clarity, only one component is shown.

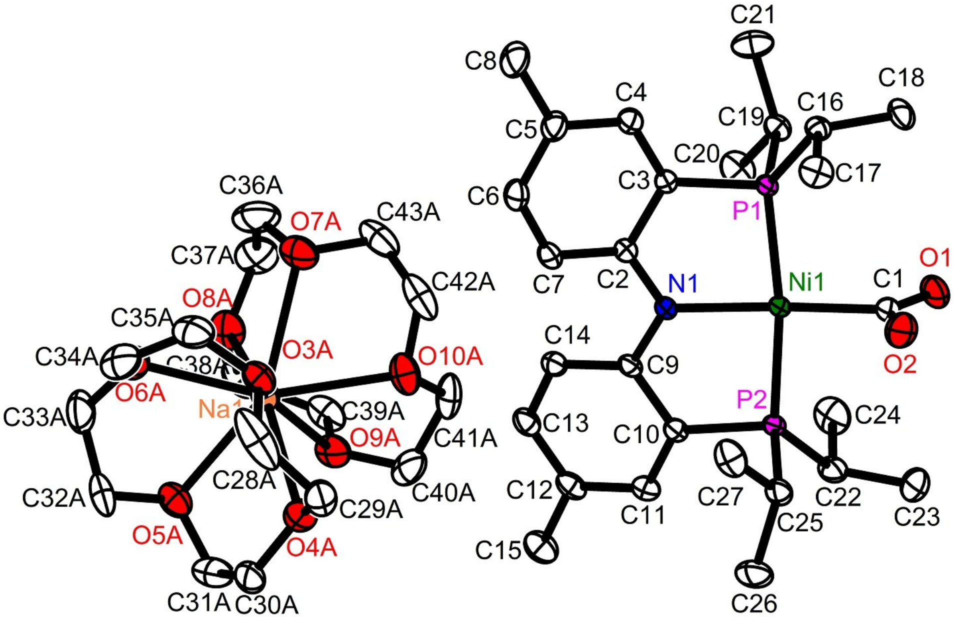

Table S1. Selected bond distances and angles for of {Na(12-C-4)$_2$}{(PNP)Ni-η^1-COO-κ*C*} (**3**) (Å and °).

Bond distance		Bond angle	
$d_{Ni1\text{-}N1}$	1.981(1)	∠N1-Ni1-C1	173.13(7)
$d_{Ni1\text{-}P1}$	2.1489(4)	∠P1-Ni1-P2	169.51(2)
$d_{Ni1\text{-}P2}$	2.1484(4)	∠N1-Ni1-P1	85.30(4)
$d_{Ni1\text{-}C1}$	1.911(2)	∠N1-Ni1-P2	85.93(4)
$d_{C1\text{-}O1}$	1.247(2)	∠Ni-C1-O1	121.6(2)
$d_{C1\text{-}O2}$	1.248(2)	∠Ni-C1-O1	109.9(1)
$d_{Ni1\text{-}O1}$	2.776(1)	∠O1-C1-O2	128.4(2)
$d_{Ni1\text{-}O2}$	2.614(1)		

Figure S6. Solid-state structure of (PNP)Ni-μ-CO_2-κ*C*:κ^2*O,O'*-Fe(PNP) (**5**). Hydrogen atoms are omitted for clarity.

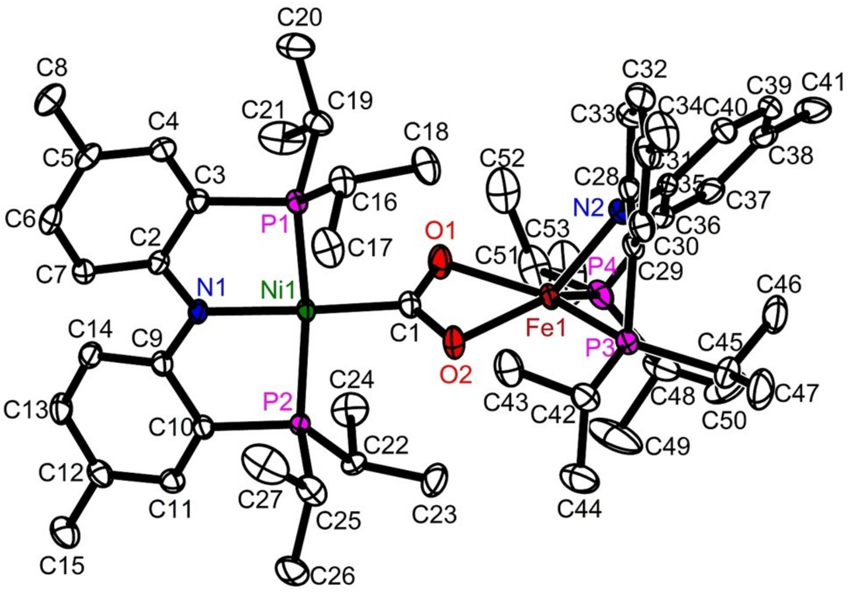

Table S2. Selected bond distances and angles for of (PNP)Ni-μ-CO_2-κ*C*:κ^2*O,O'*-Fe(PNP) (**5**) (Å and °).

Bond distance		Bond angle	
$d_{Ni1\text{-}N1}$	1.932(1)	∠N1-Ni1-C1	175.54(6)
$d_{Ni1\text{-}P1}$	2.1614(4)	∠P1-Ni1-P2	170.00(2)
$d_{Ni1\text{-}P2}$	2.1563(4)	∠N2-Fe1-O1	113.84(5)
$d_{Ni1\text{-}C1}$	1.858(1)	∠N2-Fe1-O2	140.35(5)
$d_{Fe1\text{-}N2}$	2.004(1)	∠P3-Fe1-P4	121.68(2)
$d_{Fe1\text{-}P3}$	2.5698(4)	∠O1-Fe1-P3	148.30(3)
$d_{Fe1\text{-}P4}$	2.4376(5)	∠O2-Fe1-P4	132.78(3)
$d_{Fe1\text{-}O1}$	2.204(1)	∠O2-Fe1-O1	61.13(4)
$d_{Fe1\text{-}O2}$	2.066(1)	∠O1-C1-O2	116.5(1)
$d_{C1\text{-}O1}$	1.269(2)		
$d_{C1\text{-}O2}$	1.289(2)		

Figure S7. UV-Vis spectra of {Na(12-C-4)$_2$}{(PNP)Ni-η^1-COO-κ*C*} (**3**) in THF at room temperature.

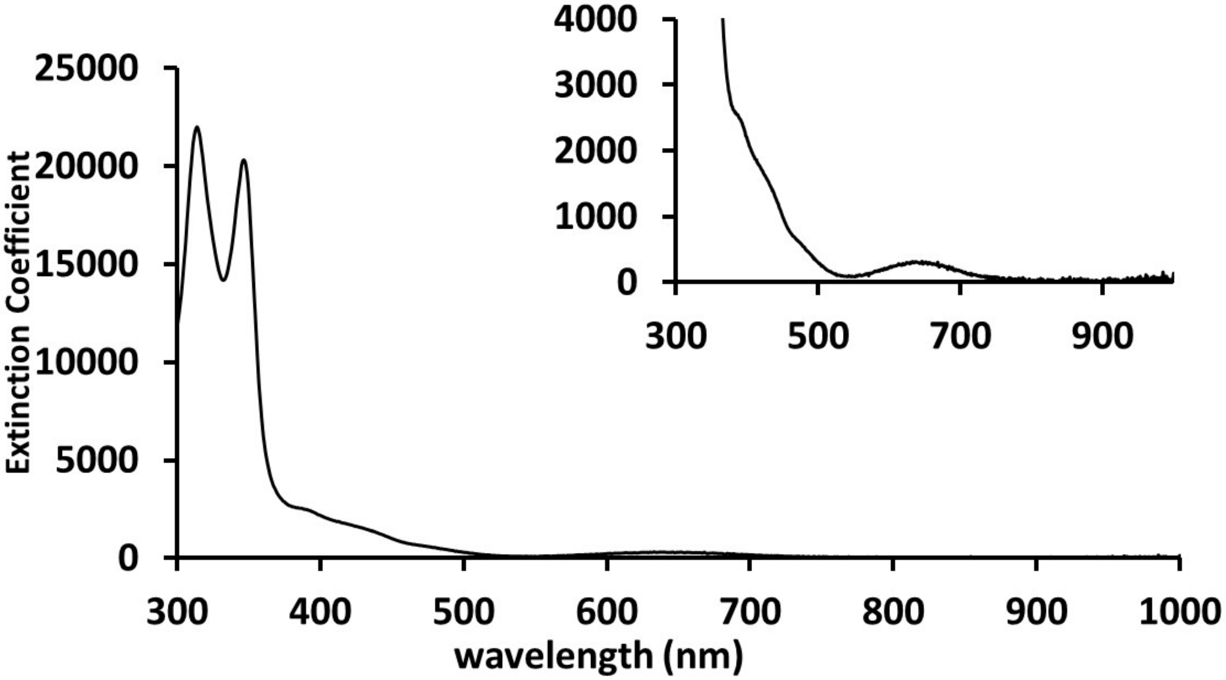

Figure S8. UV-Vis spectra of (PNP)Ni-μ-CO$_2$-κ*C*:κ^2*O,O'*-Fe(PNP) (**5**) in THF at room temperature.

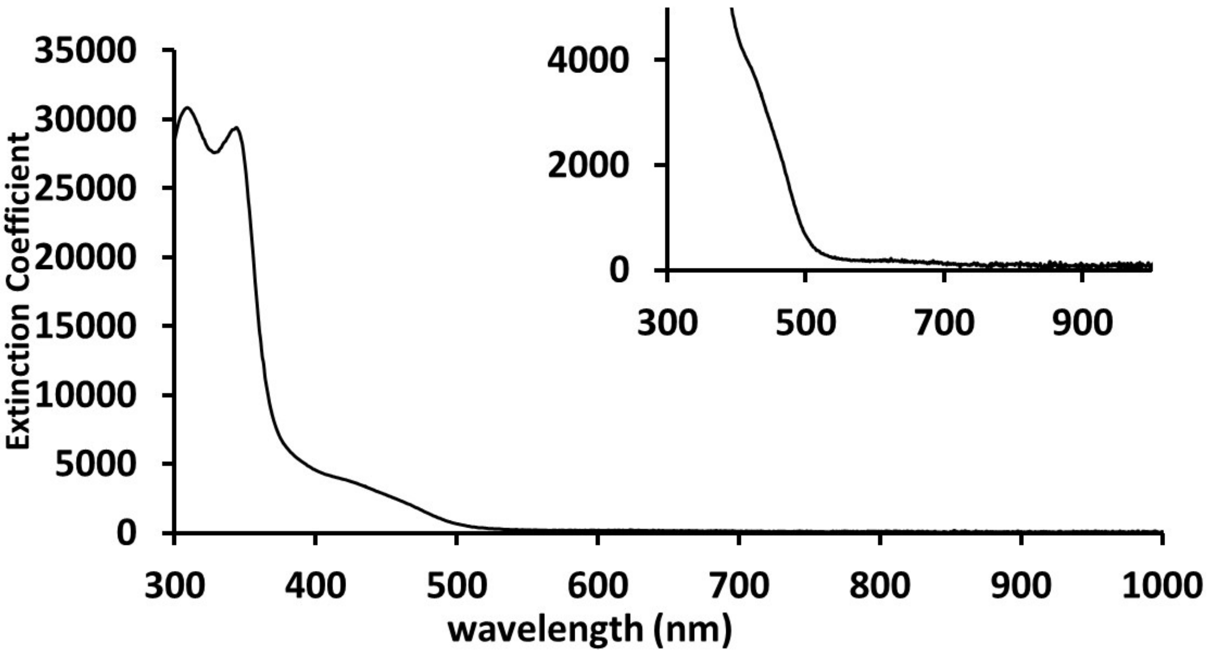

Figure S9. IR spectra of (PNP)NiCOOH (**1**, blue) and {(PNP)NiCOONa}$_2$•THF (**2**, red) and {Na(12-C-4)$_2$}{(PNP)Ni-η^1-COO-κ*C*} (**3**, green) (KBr pellet).

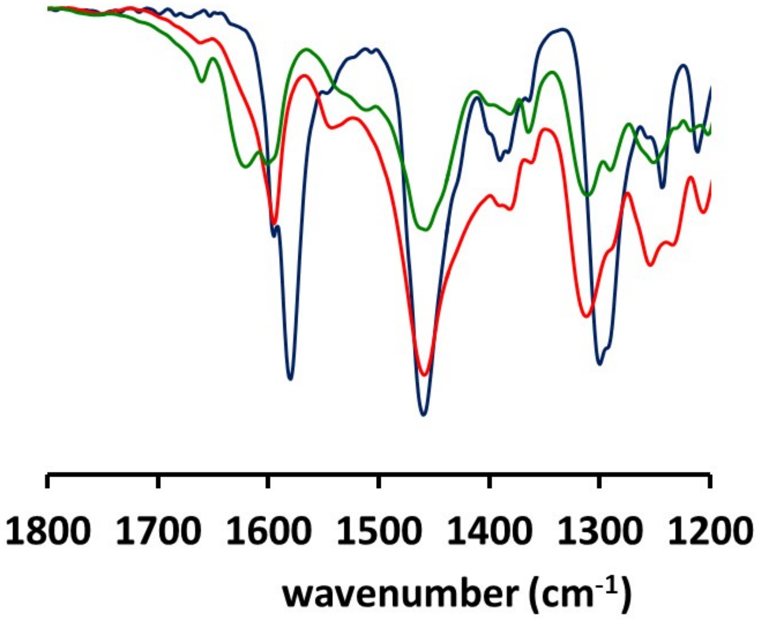

Figure S10. IR spectra of {(PNP)Ni}$_2$-μ-CO_2-κ^2*C*,*O* (**4**, blue) and (PNP)Ni-μ-CO_2-κ*C*:κ^2*O,O'*-Fe(PNP) (**5**, red) (KBr pellet).

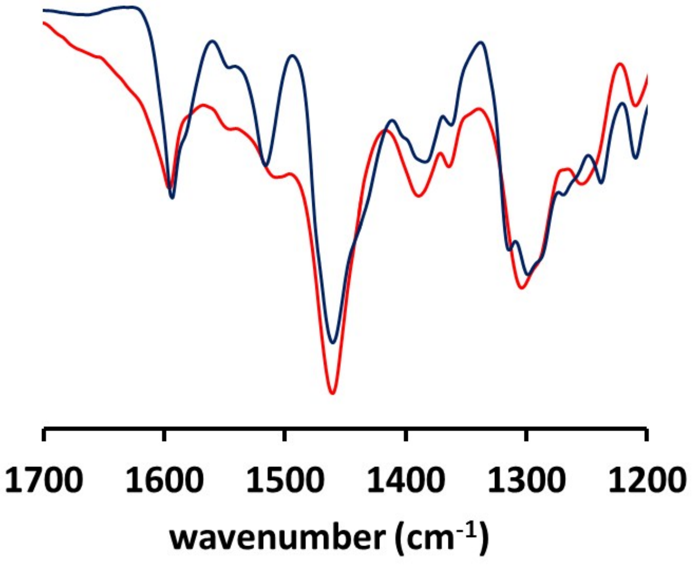

Figure S11. DFT calculated HOMO for {(PNP)NiCOO}⁻ (**3**). Lobal presentations correspond to the orbital indicated by the number with 0.06 isocontours.

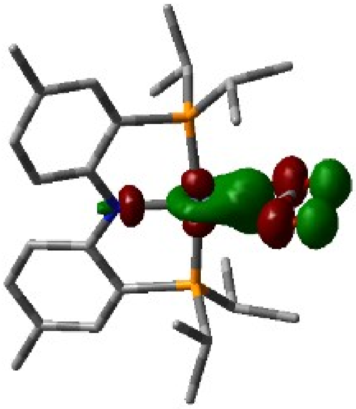

Figure S12. Electronic structures for {(PNP)NiCOO}⁻ (**3**) and (PNP)NiCOOH (**1**) derived from the single point DFT calculations; energies in eV.

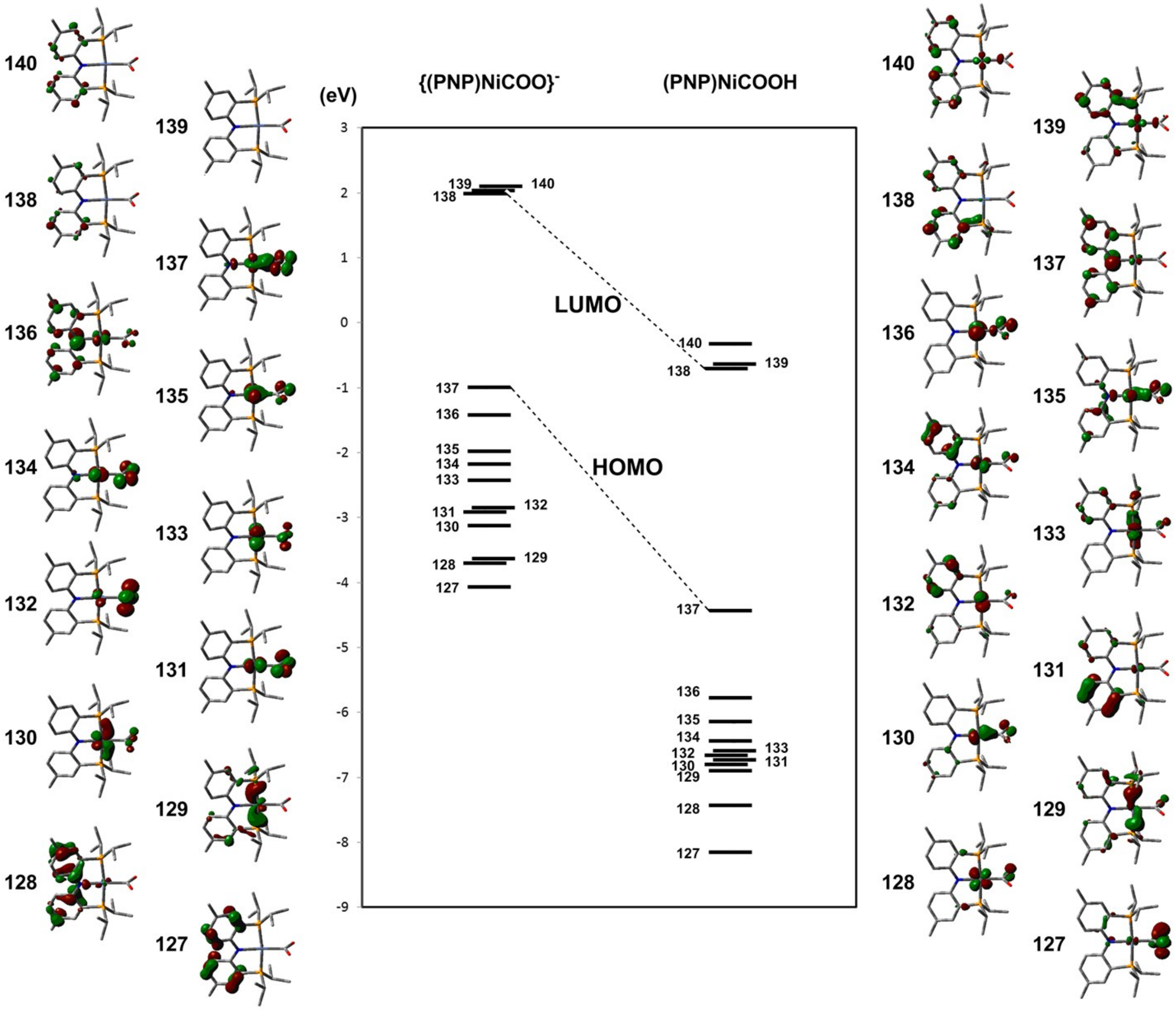

Table S3. Selected Wiberg bond indices for (dtbpe)Ni(CO_2), ($PP^{Me}P$)Ni(CO_2), {(PNP)Ni-η¹-COO-κ*C*}⁻ (**3**), (PNP)NiCOOH (**1**) and (PNP)Ni-μ-CO_2-κ*C*:κ²*O,O'*-Fe(PNP) (**5**).

	(dtbpe)Ni(CO_2)	($PP^{Me}P$)Ni(CO_2)	**3**	**1**	**5**
Ni-C	0.5766	0.5286	0.6143	0.6834	0.6277
Ni-O	0.4300	0.3117	0.1679<br>0.1358	0.1032<br>0.0412	0.0798<br>0.0644
O-Fe	-	-	-	-	0.2397<br>0.1814
C-O	1.6927<br>1.4080	1.6384<br>1.4701	1.5112<br>1.4949	1.6342<br>1.0548	1.3993<br>1.2933

Figure S13. Mülliken atomic spin density plot derived from the single-point DFT calculation of (PNP)Ni-μ-CO_2-κ*C*:κ²*O,O'*-Fe(PNP) (**5**). Lobal representations correspond to the spin density by the number with 0.004 isocontours.

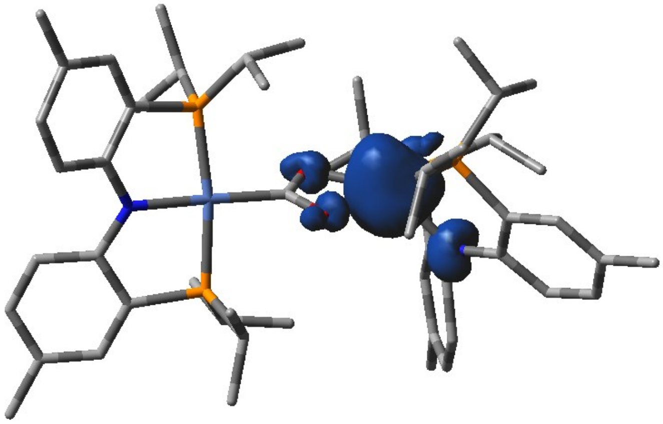

Ni: –0.013; N1: –0.003; P1: –0.010; P2: 0.029;

Fe: 4.275; N2: 0.095 P3: –0.186; P4: –0.232;

C1: –0.175; O1: 0.040; O2: 0.062

References

1 C. Yoo, J. Kim and Y. Lee, *Organometallics*, 2013, **32**, 7195.

2 D. Adhikari, F. Basuli, H. Fan, J. C. Huffman, M. Pink and D. J. Mindiola, *Inorg. Chem.*, 2008, **47**, 4439.

3 SMART, Version 5.0; data collection software, Bruker AXS, Inc., Madison, WI, USA, 1998.

4 SAINT, Version 5.0; data integration software, Bruker AXS, Inc., Madison, WI, USA, 1998.

5 G. M. Sheldrick, SADABS: program for absorption correction with the Bruker SMART system, Universität Göttingen, Göttingen, Germany, 1996.

6 G. M. Sheldrick, SHELXTL, Version 6.1, Bruker AXS, Inc., Madison, WI, USA, 2000.

7 (a) D. F. Evans, *J. Chem. Soc.*, 1959, 2003; (b) S. K. Sur, *J. Magn. Reson.*, 1989, **82**, 169.

8 (a) J. S. Anderson, V. M. Iluc and G. L. Hillhouse, *Inorg. Chem.*, 2010, **49**, 10203; (b) Y.-E. Kim, J. Kim and Y. Lee, *Chem. Commun.*, 2014, **50**, 11458.

9 M. J. Frisch, G. W. Trucks, H. B. Schlegel, G. E. Scuseria, M. A. Robb, J. R. Cheeseman, G. Scalmani, V. Barone, B. Mennucci, G. A. Petersson, H. Nakatsuji, M. Caricato, X. Li, H. P. Hratchian, A. F. Izmaylov, J. Bloino, G. Zheng, J. L. Sonnenberg, M. Hada, M. Ehara, K. Toyota, R. Fukuda, J. Hasegawa, M. Ishida, T. Nakajima, Y. Honda, O. Kitao, H. Nakai, T. Vreven, J. A. Montgomery, Jr., J. E. Peralta, F. Ogliaro, M. Bearpark, J. J. Heyd, E. Brothers, K. N. Kudin, V. N. Staroverov, R. Kobayashi, J. Normand, K. Raghavachari, A. Rendell, J. C. Burant, S. S. Iyengar, J. Tomasi, M. Cossi, N. Rega, J. M. Millam, M. Klene, J. E. Knox, J. B. Cross, V. Bakken, C. Adamo, J. Jaramillo, R. Gomperts, R. E. Stratmann, O. Yazyev, A. J. Austin, R. Cammi, C. Pomelli, J. W. Ochterski, R. L. Martin, K. Morokuma, V. G. Zakrzewski, G. A. Voth, P. Salvador, J. J. Dannenberg, S. Dapprich, A. D. Daniels, Ö. Farkas, J. B. Foresman, J. V. Ortiz, J. Cioslowski and D. J. Fox, Gaussian 09, Revision A.02, Gaussian, Inc., Wallingford, CT, 2009.
